# Effects of Extreme-Duration Heavy Load Carriage on Neuromuscular Function and Locomotion: A Military-Based Study

**DOI:** 10.1371/journal.pone.0043586

**Published:** 2012-08-22

**Authors:** Jordane G. Grenier, Guillaume Y. Millet, Nicolas Peyrot, Pierre Samozino, Roger Oullion, Laurent Messonnier, Jean-Benoît Morin

**Affiliations:** 1 Laboratory of Exercise Physiology (EA4338), University of Lyon, Saint-Etienne, France; 2 Safran Group, Sagem, Land Warfare, Massy, France; 3 University of La Reunion, UFR SHE, CURAPS-DIMPS (EA4075), Reunion Island, France; 4 Laboratory of Exercise Physiology (EA4338), University of Savoie, Le Bourget du Lac, France; 5 Sports Medicine and Myology Unit, Bellevue Hospital, University Hospital Center, Saint-Etienne, France; Universidad Europea de Madrid, Spain

## Abstract

Trekking and military missions generally consist of carrying heavy loads for extreme durations. These factors have been separately shown to be sources of neuromuscular (NM) fatigue and locomotor alterations. However, the question of their combined effects remains unresolved, and addressing this issue required a representative context.

**Purpose:**

The aim was to investigate the effects of extreme-duration heavy load carriage on NM function and walking characteristics.

**Methods:**

Ten experienced infantrymen performed a 21-h simulated military mission (SMM) in a middle-mountain environment with equipment weighing ∼27 kg during battles and ∼43 kg during marches. NM function was evaluated for knee extensors (KE) and plantar flexors (PF) pre- and immediately post-SMM using isometric maximal voluntary contraction (MVC) measurement, neural electrical stimulation and surface EMG. The twitch-interpolation method was used to assess central fatigue. Peripheral changes were examined by stimulating the muscle in the relaxed state. The energy cost, mechanical work and spatio-temporal pattern of walking were also evaluated pre−/post-SMM on an instrumented treadmill in three equipment conditions: Sportswear, Battle and March.

**Results:**

After the SMM, MVC declined by −10.2±3.6% for KE (*P*<0.01) and −10.7±16.1% for PF (*P* = 0.06). The origin of fatigue was essentially peripheral for both muscle groups. A trend toward low-frequency fatigue was detected for KE (5.5%, *P* = 0.08). These moderate NM alterations were concomitant with a large increase in perceived fatigue from pre- (rating of 8.3±2.2) to post-SMM (15.9±2.1, *P*<0.01). The SMM-related fatigue did not alter walking energetics or mechanics, and the different equipment carried on the treadmill did not interact with this fatigue either.

**Conclusion:**

this study reports the first data on physiological and biomechanical consequences of extreme-duration heavy load carriage. Unexpectedly, NM function alterations due to the 21-h SMM were moderate and did not alter walking characteristics.

**Clinical Trial Registration:**

**Name:** Effect of prolonged military exercises with high load carriage on neuromuscular fatigue and physiological/biomechanical responses. Number: NCT01127191.

## Introduction

Outdoor activities requiring self-sufficiency, specific gear and/or involving bivouac (e.g. trekking and military missions) are characterized by the carriage of considerable equipment and supplies. This specific feature implies high-to-severe conditions of locomotion [1], [2] and requires high levels of metabolic and mechanical energy from the carrier, even for short-term walking [3], [4], [5], [6]. In addition, these walking efforts are generally prolonged for hours or even days and can be associated with sleep rhythm disturbance or deprivation, caloric restriction, as well as environmental and psychological stresses, especially in the military context [7], [8], [9]. Taken together or separately, these factors have deleterious physiological consequences [10], [11] and may induce neuromuscular (NM) fatigue.

NM fatigue is defined as an exercise-related decrease in the maximal strength or power of a muscle, whether or not the task can be sustained [12]. Fatigue potentially involves processes at all levels of the motor pathway from the motor cortex to skeletal muscle [13]. Classically, alterations of neuromuscular function due to fatigue are classified as central (neural) or peripheral (muscular) in origin. Central fatigue corresponds to a failure of the central nervous system to drive the motoneurons adequately and appears with exercise [14] while peripheral fatigue may involve all processes located distal to the neuromuscular junction/sarcolemma, in particular the fatigue-induced alterations in excitation–contraction coupling [15]. Central and peripheral origins are mutually dependent since recruitment of motoneurons depends on the descending drive from supraspinal sites and central drive is controlled through a combination of factors including excitatory and inhibitory reflex inputs from different peripheral afferents. Fatigue is known to depend on exercise duration and intensity, and type of muscle contraction [16], [17].

Previous studies have investigated the effects of exercises with heavy load carriage of short-to-medium duration on NM function. Clarke et al. [18] have reported decreases in trunk, knee and ankle flexor/extensor maximal voluntary contraction (MVC) in soldiers after ∼3-h walking (12.1 km at 4 km.h^−1^) while carrying loads up to 27 kg. More recently, Blacker et al. [19] showed that the carriage of a 25-kg pack during 2-h treadmill walking induced a 15% loss in knee extensor (KE) MVC and was associated with moderate central and peripheral fatigue. Furthermore, slight low-frequency fatigue (LFF, which has been linked to excitation-contraction coupling failure and muscular damage [20]) was detected in this study for both level and downhill walking [19].

Other studies have investigated the NM consequences of exercises without load carriage (or with light equipment) but of extreme duration. Very large KE and plantar flexor (PF) MVC declines were observed in ultra-marathon runners after a 24-h treadmill run (−41% and −30%, respectively [21]) and a ∼40-h mountain ultra-marathon (−35% and −39%, respectively [22]). In both studies, MVC declines were associated with large central activation deficits (depending on the muscle group tested) and peripheral fatigue. LFF was also observed after the mountain ultra-marathon, likely due to long downhill sections (∼9 500 m of total negative elevation) that involved intense eccentric muscular actions known to induce muscular damage [22], [23].

To date, the question of the combined effects of heavy load carriage and exercise of extreme duration on NM fatigue remains however unresolved. Since military efforts naturally and severely combine these two features [9], we thought it justified and relevant to use a military mission context to address this question. Indeed, beyond their scientific novelty, such data obtained in a real-world context could be applied to strategic planning and used in the military theater (e.g. management of forces during actual missions). Therefore, the main purpose of the present study was to investigate the effects of a prolonged mission with heavy military load carriage on the NM fatigue of two major muscle groups involved in human walking and load carriage [24]. For this purpose, the KE and PF NM function of experienced infantrymen was assessed before and after a 21-h simulated military mission (SMM) specifically designed to represent the current operational reality.

In addition, in view of the existing links between NM fatigue, load carriage and the risks of slips, falls or injuries during locomotion [2], [24], [25], [26], [27], [28], this study design allowed us to investigate the effects of extreme-duration heavy load carriage on walking mechanics and energetics. Indeed, although acute consequences of load carriage have been widely considered [3], [4], [5], [6], very little is known about the effects of fatigue and its potential interaction with load carriage on walking characteristics. Moreover, the few studies that have hitherto explored similar issues mostly used fatiguing laboratory protocols (e.g. treadmill run [27], [29], [30], [31]), not real exercises in the field, and this may limit the representativity of their observations [32]. Therefore, the secondary aim of the present study was to investigate the consequences of this 21-h SMM on the energy cost, mechanical work and spatio-temporal parameters of walking.

Concerning the primary aim of the present study, we hypothesized that both central and peripheral NM function alterations would be larger after the SMM than after loaded-walking exercises of intermediate duration, i.e. from 2 to 3 h [18], [19]. This is because of (i) the much longer exercise duration, (ii) the heavier loads carried, and (iii) the additional stressors inherent to the mission-related effort (e.g. sleep rhythm and comfort disturbances, hilly terrain). However, NM function changes of lower magnitude than after ultra-marathon runs [21], [22] were expected (especially at the peripheral level) because, contrary to running, walking is not associated with repeated impacts or intense muscular eccentric actions [33], [34]. Regarding the secondary aim of this study, we hypothesized that walking characteristics would change after the 21-h SMM, especially the mechanical parameters [30], [31] that have been shown to be more discriminating than energetical ones at moderate walking speed [4].

## Methods

### Subjects and Ethics Statement

Ten males volunteered to participate in this study after being informed about the procedure and risks associated with the protocol. They were all involved in regular physical activities (5.4±2.7 hours per week) and were not presenting recent muscular, joint or bone disorders or receiving any medication that could interfere with their NM responses or walking pattern or influence their energetic metabolism. The subjects were recently-retired infantrymen (seven from the French Foreign Legion) with a career of 14.1±8.3 years and had extensive high-load carriage and military experience. Their anthropometrical and physiological characteristics were as follows: age: 38.9±8.9 yrs, height: 177±5 cm, mean leg length: 90.9±3.6 cm, body mass (BM): 82.9±9.3 kg, BM index: 26.7±2.2 kg.m^−2^, body fat percentage: 19.4±3.1%, maximal heart rate (HR_max_): 190±17 bpm, absolute maximal oxygen uptake (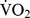
_max_): 3.53±0.36 L.min^−1^, 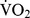
_max_ relative to BM: 42.3±5.2 mL.kg^−1^.min^−1^.

Written informed consent was obtained from the subjects, and the study was conducted according to the Declaration of Helsinki. The protocol was approved by the local ethics committee (Comité de Protection des Personnes, Sud-Est 1, France) and registered at http://clinicaltrial.gov (reference: NCT01127191).

### Protocol Overview

The subjects were included in the study one week before the beginning of the specific research protocol. Inclusion sessions consisted of (i) a complete medical examination with anthropometric data collection, (ii) a standardized incremental maximal aerobic test, and (iii) a complete familiarization with the different devices/protocols used in the experimentation (see details in the section *subject characterization and familiarization* below).

**Figure 1:**
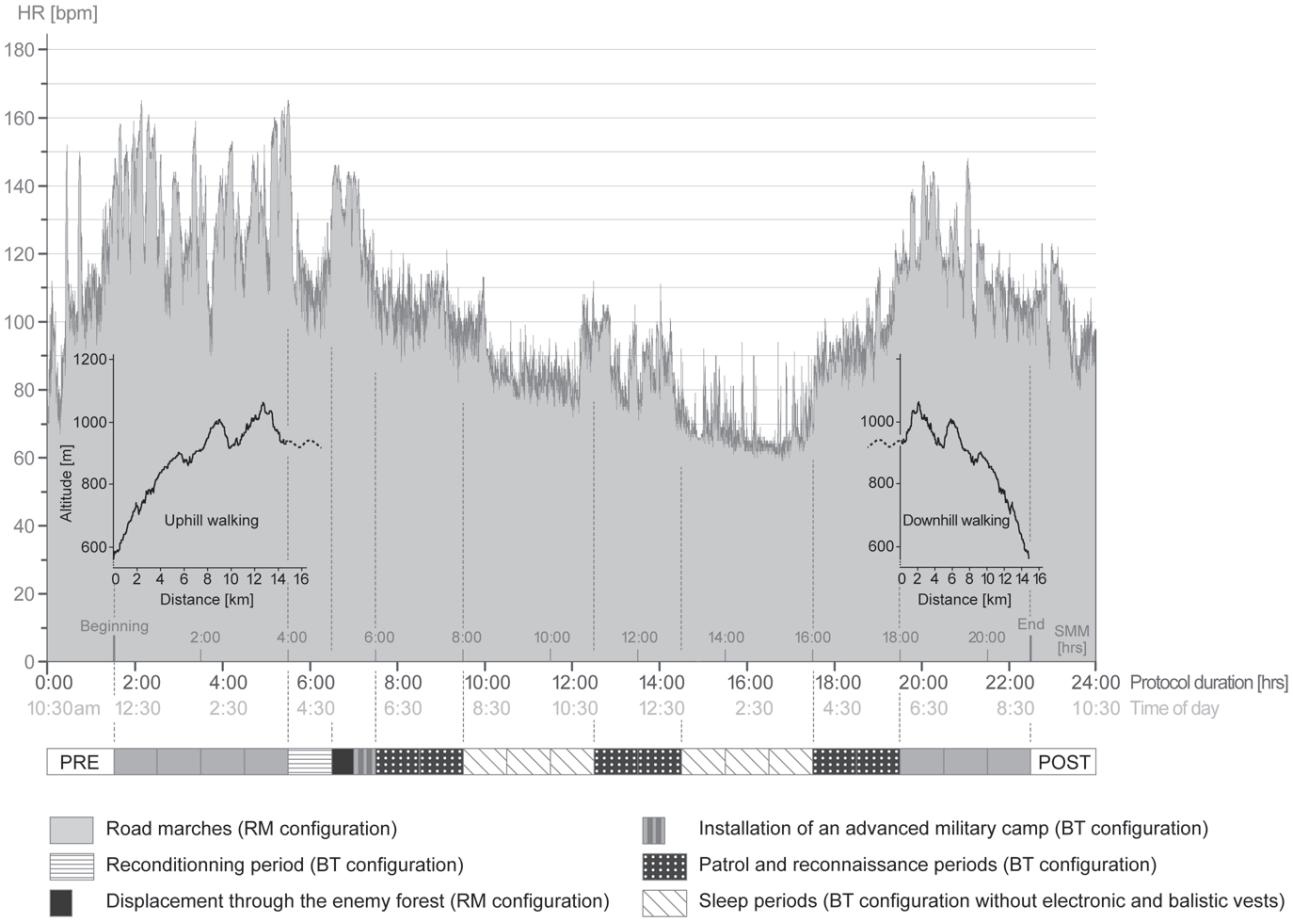
Typical heart rate (HR) of a subject throughout the protocol. Figure includes the 21-h simulated military mission (SMM) and the pre-SMM (PRE) and post-SMM (POST) measurement sessions. Altitude, chronology and equipment conditions are inserted on and under the HR graph as indicative data. BT: battle equipment (27.4±1.1 kg corresponding to 33.4±2.6% of the subjects’ BM), RM: road march equipment (42.9±1.4 kg, corresponding to 52.2±4.2% BM).

The specific research protocol lasted 24 h and consisted of two (pre/post) 90-min laboratory measurement sessions separated by the 21-h SMM. Heart rate was the only parameter monitored continuously throughout the protocol using telemetric cardiofrequencemeters (Polar RS800CX, Polar Electro Oy, Kempele, Finland). Chronologically, the pre-SMM session (PRE) began with an evaluation of the subjects’ instant rating of perceived fatigue (RPF), and a 6-min moderate cycling warm-up immediately followed by a standardized NM function evaluation (see details in the sections relating to *fatigue assessment* below). After 5 min of rest, expired gases were collected over 10 min of unloaded standing. Then the subjects performed three 3-min level-walking trials at 4 km.h^−1^ on an instrumented treadmill, during which walking energetics and mechanics were assessed in three loading conditions (i.e. one condition per trial, see details in the sections *equipment/load characteristics* and *walking assessment* below). Before starting the SMM at 12∶00 am, the subjects checked their equipment and supplies. The 21-h SMM was performed in a low- to middle-mountain environment close to the laboratory (see details in the section *SMM characteristics* and figure 1 below). After the SMM, the subjects immediately performed the post-SMM session (POST). POST began with an evaluation of instant RPF and rating of perceived exertion regarding the entire SMM (RPE). Then, after taking off their equipment, the subjects performed the NM function evaluation within 9±3 min after the SMM. Finally, the course of POST (i.e. unloaded standing gas collection and walking assessments on the treadmill in three loading conditions) was identical to that of PRE.

For logistical reasons (duration of PRE and POST, follow-up and safety of the subjects during the SMM, etc.), the subjects were divided into two subgroups (n = 5) and performed the protocol two days apart. Similarly, in order to avoid a period of rest between the end of the SMM and the NM function evaluations at POST, the subjects in a same subgroup performed the protocol at 20-min intervals.

### Subject Characterization and Familiarization

During the inclusion sessions, the subjects were first examined by the same medical doctor that also performed anthropometric data collection. Stature was measured to the nearest 0.5 cm using a standardized wall-mounted height board. Leg length was measured from the great trochanter to the floor in a standing position with a tape measure. BM was measured to the nearest 0.1 kg with subjects standing in undergarments without shoes on a mechanical column weighing scale (Bascule type 286, Chollet, La Talaudière, France). BM Index was calculated as BM divided by height (in m) squared. Body fat percentage was estimated using skinfold thickness values and Durnin & Womersley standard equations [35]. Skinfold thickness values were measured to the nearest millimeter in triplicate at the biceps, triceps, subscapular, and suprailiac points on the left and right side of the body using a Harpenden skinfold caliper (British Indicators, West Sussex, UK). At each of these four points, the mean value for the six skinfold thicknesses was calculated.

After this examination, the subjects performed an incremental running maximal test on a level treadmill. This test consisted of progressive 3-min stages separated by 1 min of rest. Running speed was 10 km.h^−1^ at the first stage and the increment of speed was 1.5 km.h^−1^ per stage until exhaustion. Heart rate (HR) was monitored continuously using a three-channel electrocardiogram (Cardiotest EK51, Hellige GMBH, Freiburg, Germany). Expired gases were collected during the last 30 s of each 3-min stage to determine oxygen consumption (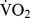). The subjects breathed through a two-way nonrebreathing valve (series 2700; Hans Rudolph, Kansas City, MO) connected to a three-way stopcock leading to a 100-L Douglas bag. The volume of the expired gas was measured by means of a Tissot spirometer (Gymrol, Roche-la-Molière, France), and fractions of expired gases were determined with a paramagnetic O_2_ analyzer (cell 1155B; Servomex, Crowborough, England) and an infrared CO_2_ analyzer (Normocap Datex, Helsinki, Finland). The analyzers were calibrated with mixed gases of known composition. HR_max_ and 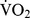
_max_ corresponded to the highest values obtained at steady state during the last running stage.

Particular attention was paid to familiarize the subjects with the devices/protocols used during the specific experimentation, especially the MVC and electrical stimulation of the KE and PF muscles. The subjects repeated trials of the procedures until the results were reproducible.

### Equipment/load Characteristics

For a detailed description and illustration of the equipment, the reader should refer to a recent article from our research group [4]. Briefly, during the laboratory walking trials, three equipment conditions were tested, a Sportswear condition taken as reference (SP, mass ≤1 kg, corresponding to ≤1.4% of the subjects’ BM) and two configurations of the new French infantry combat system (FELIN, Sagem, France) without rifle: Battle equipment (BT, 22.4±1.1 kg, 27.2±1.9% BM) and Road March equipment (RM, 37.9±1.4 kg, 46.1±3.6% BM, corresponding to BT with an additional backpack).

During the SMM, both military BT and RM equipment were carried alternately (according to the military actions performed, e.g. RM while marching and BT while patrolling, see figure 1) with an additional demilitarized rifle weighting 5.01 kg (FA-MAS FELIN, France) to create conditions comparable to those found in the military theater. The total loads carried during the SMM were therefore 27.4±1.1 kg in BT and 42.9±1.4 kg in RM, corresponding to 33.4±2.6% BM and 52.2±4.2% BM respectively. BT and RM configurations were designed to represent and meet the common necessities of a prolonged patrol and reconnaissance mission. Each participant was familiarized with all of the equipment tested prior to the experiment.

### Simulated Military Mission (SMM) Characteristics

An overview of the 21-h SMM design, intensity and integration in the general protocol is given in figure 1. The SMM was specifically designed by former officers to represent a typical prolonged patrol and reconnaissance mission. It began with a 4-h road march to simulate quietly approaching a hostile zone represented by a fragment of forest located in a middle-mountain environment 15 km away from the laboratory (uphill walking, 570 m of positive and 240 m of negative elevation change, mean walking speed of ∼4 km.h^−1^). On arrival near the hostile zone, the subjects had a 1-h period to rest and ration. Each subject had a French war ration (“RCIR”; 3200 kcal, protein: 13%, lipid: 32%, carbohydrate: 55%) in his pack to provide sustenance over the entire SMM. After this reconditioning period, the subjects moved through the enemy forest (∼30 min) in order to find and install (∼30 min) an advanced camp. The objective of the following 2-h recon period was to progressively secure the zone that surrounded the camp while approaching a strategic enemy lane for observation. During the night hours, the subjects were equipped with night vision devices. Moreover, during the recon periods of the SMM, experimenters drove and moved through the forest to simulate enemy activity. The subjects were also told to try and intercept the experimenters as they tried to reach the advanced camp, just as enemies would do. After the aforementioned 8 h of various activities, the subjects were allowed two 3-h sleeping periods separated by another 2-h recon period. Sleeping periods were set and imposed according to the common turnover observed in missions and in previous reports [9]. Then one last 105-min recon was performed prior to a 15-min period during which the subjects gathered their equipment prior to walking back to the laboratory. The return road march was the exact reverse of the first one (downhill walking, 570 m of negative and 240 m of positive elevation change) except it was performed in 3 h because of the major negative slope (mean walking speed of ∼5.5 km.h^−1^).

### Fatigue Assessment: Neuromuscular Function

#### Experimental materials, preparation and setting

In the present study, we used the exact same (i) ergometers, positioning and instructions to subjects, (ii) electrical stimulation system and neural detection procedure, (iii) electrode models, electromyographic recording system and skin preparation procedure as in a recent study from our research group [22].

Maximal voluntary contractions: the subjects were strongly encouraged during all the MVCs. For the KE testing, the subjects were seated in the frame of a Cybex II (Ronkonkoma, NY) and Velcro straps were strapped across the chest and hips to avoid lateral and frontal displacements. Subjects were also instructed to grip the seat during the MVC to further stabilize the pelvis. The KE muscular mechanical response was recorded with a strain gauge (SBB 200 Kg, Tempo Technologies, Taipei, Taiwan) located at the level of the external *malleolus*. All measurements were taken from the subject’s right leg with the knee and hip flexed at 90 degrees from full extension. PF muscles were tested with an instrumented pedal (CS1060 300 Nm, FGP Sensors, Les Clayes Sous Bois, France). For the PF testing, the subjects were seated in the frame of a Cybex II similar to that used for KE. Velcro straps were also strapped across the chest and hips to avoid lateral and frontal displacements, and across the forefoot to limit heel lift during the MVC. The hip, knee and ankle angles were set at 90 degrees from full extension.

Electrical stimulation: after femoral (for KE) and posterior tibial nerve (for PF) detection with a ball probe cathode pressed into the femoral triangle and the popliteal fossa, respectively, electrical stimulation was applied percutaneously to the motor nerve via a self-adhesive electrode pressed manually (10-mm diameter, Ag-AgCl, Type 0601000402, Contrôle Graphique Medical, Brie-Comte-Robert, France). The anode, a 10×5 cm self-adhesive stimulation electrode (Medicompex SA, Ecublens, Switzerland), was located either in the gluteal fold (for KE) or on the *patella* (for PF). A constant current stimulator (Digitimer DS7A, Hertfordshire, United Kingdom) was used to deliver a square-wave stimulus of 1000-µs duration with maximal voltage of 400 V. The stimulation intensity (70.6±15.6 mA at PRE and 72.4±16.9 mA at POST in KE, and 64.6±17.2 mA at PRE and 65.3±15.1 mA at POST in PF) was determined from maximal mechanical response to single twitch delivered to the relaxed muscle. This stimulation intensity was supramaximal and corresponded to 130% of the optimal intensity.

Electromyographic recordings: the EMG signals of the right *vastus lateralis* (*VL*) and *soleus* (*SOL*) were recorded using bipolar silver chloride surface electrodes of 10-mm diameter (Type 0601000402, Contrôle Graphique Medical, Brie-Comte-Robert, France) during the MVCs and electrical stimulation. The recording electrodes were taped lengthwise on the skin over the muscle belly, with an interelectrode distance of 25 mm. The position of the electrodes was marked directly on the skin with a permanent marker so that they could be placed in the exact same position before and after the SMM. The reference electrode was on the *patella* (for *VL* EMG) or *malleolus* (for *SOL* EMG). Low impedance (Z<5 kΩ) at the skin-electrode surface was obtained by abrading the skin with fine sand paper and cleaning with alcohol. EMG data were recorded with PowerLab system (16/30 - ML880/P, ADInstruments, Bella Vista, Australia) with a sampling frequency of 2000 Hz. The EMG signal was amplified with octal bio-amplifier (Octal Bioamp, ML138, ADInstruments), with a bandwidth frequency ranging from 5 to 500 Hz (input impedance = 200 MΩ, common mode rejection ratio = 85 dB, gain = 1000), transmitted to the computer and analyzed with LabChart 6 software (ADInstruments).

#### Experimental procedure

The present procedure was also identical to that used in our recent study [22]. Briefly, the NM function evaluation consisted of determining the isometric KE and PF MVC. During MVC, when the torque had reached a plateau, a high-frequency (100 Hz) doublet was superimposed on the contracted muscle. Finally, ∼2 s after the end of the MVC, evoked stimuli consisting of a high-frequency (100 Hz) doublet, a low-frequency (10 Hz) doublet and a single twitch were delivered to the relaxed muscle in a potentiated state. This experimental set (MVC with superimposed doublet + evoked stimuli to the relaxed muscle) was repeated three times for both muscle groups with recovery of 1 min between repetitions.

#### Experimental variables and data analysis

M-wave: for both *VL* and *SOL*, M-wave peak-to-peak amplitude (in mV) and duration (in ms) were averaged from the EMG data from the three single potentiated twitches delivered to the relaxed muscle. M-wave characteristics provided information on action potential propagation.

Mechanical responses to nerve stimulation: for both KE and PF, the amplitude of the potentiated high-frequency doublet (PDb100), the ratio of paired-stimulus peak forces at 10 Hz to 100 Hz (Db10∶100) and the amplitude of the potentiated peak twitch (Pt) were averaged from the values computed during the three experimental sets. Potentiated twitch contraction time (CT, in ms) and half-relaxation time (HRT, in ms) were also determined and were also calculated as the mean values of the three single twitches. Mechanical responses to nerve stimulation were used to determine the extent and origin of peripheral fatigue. In particular, Db10∶100 was used to assess LFF [36].

Maximal voluntary contraction and maximal voluntary activation level: the highest value of the three MVC was determined for both KE (in N) and PF (in Nm). MVC provided a global index of fatigue. Activation level (%VA) was calculated as follows:
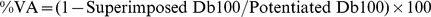



%VA was used as an indicator of central fatigue. Finally, the root mean square (RMS) values of the *VL* and *SOL* EMG activity were calculated during the best MVC trial over a 0.5-s period after the MVC had reached a plateau and before the superimposed stimulus was delivered. This RMS value was then normalized to the maximal peak-to-peak amplitude of the M-wave to obtain RMS.M^−1^. The latter variable provided complementary information about central fatigue.

### Fatigue Assessment: Perceived Fatigue and Global Exertion

RPE was measured at POST only using the 6 to 20 Borg scale [37]. The subjects were asked to quantify the exertion characterizing the entire SMM. RPF was measured at PRE and POST, using the same 6 to 20 Borg scale applied to the sensation of fatigue. The subjects were asked to quantify their instant sensation of general fatigue. RPE and RPF were administered individually to subjects by the same experimenter for all subjects.

### Walking Assessment

The three equipment conditions, SP, BT and RM, were performed in a randomized and counterbalanced order, and trials were separated by 5 to 10 min during which subjects rested and changed their equipment. The 4 km.h^−1^ walking speed was chosen for its economical feature in normal adult locomotion [3], [38] and its consistency with the average walking speed currently used during military missions and experimentations [39]. Finally, rifle carriage was excluded to allow comparisons of our results with non-military studies since walking kinetics are altered by a limitation of the arm swing during rifle carriage [40]. Complete details of the physiological and biomechanical methods employed have been recently presented [4]. Thus, only the main principles and parameters are summarized below.

### Walking Energetics

Energetic data were obtained from indirect calorimetry (Douglas bag method, *vide supra*). Expired gases were collected during the last 30 s of each 3-min walking trial. Unloaded standing metabolic rate and gross metabolic rate of walking (both in W) were determined from the steady-state 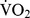 and 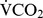 using Brockway’s standard equation [41]. The metabolic rate measured during unloaded standing was subtracted from all gross walking values to compute the net metabolic rate (in W) [5]. Gross and net metabolic rates (in W) were divided by walking speed (in m.s^−1^) to obtain the gross and net energy costs of walking (*C*_W_, in J.m^−1^). Gross and net *C*_W_ were also divided by the total mass in motion on the treadmill (TM, in kg), i.e. subject plus equipment, to obtain mass-relative gross and net *C*_W_ (*C*_W.TM_, in J.kg^−1^.m^−1^).

### Walking Mechanics

Walking mechanics were analyzed using an instrumented 3-D force treadmill (ADAL, HEF Tecmachine, Andrézieux-Bouthéon, France) consisting of two left-right frames and belts allowing separate measurements of the left- and right-foot ground reaction forces (for complete description and validation, see ref. [42]). Parameters were recorded over 20 s, 1.5 min after the beginning of each trial in order to ensure the stabilization of the gait pattern and avoid disturbances from the metabolic measurements. All data were sampled at 200 Hz and low-pass filtered at 30 Hz. Mechanical analyses were performed over five consecutive strides, one stride being defined as the period between two consecutive right heel strikes. Mechanical parameters were computed for each stride and then averaged to describe a typical mean stride.

Spatio-temporal parameters of walking were calculated from vertical ground reaction force signals. A duty factor (in %) was calculated as the ratio of stance duration to stride duration, and double support duration (in %) as the ratio of stance duration to stride duration. Finally, step frequency (in Hz) was computed as: (stride duration/2)^−1^.

Kinetic parameters were computed from vertical, antero-posterior, and medio-lateral ground reaction forces. The external mechanical work (*W*_ext_, in J.m^−1^), i.e. the work done by the muscles to lift and accelerate the center of mass (COM), was calculated according to Cavagna’s standard method [43]. *W*_ext_ was also normalized by the total moving mass (TM) to obtain mass-relative *W*_ext_ (*W*_ext.TM_, in J.kg^−1^.m^−1^). The inverted pendulum recovery of mechanical energy of the COM (in %) was calculated according to Schepens et al. [44]. The internal work done during the double contact phase (*W*_int,dc_, in J.m^−1^), i.e. the work done by one leg against the other during the transfer from one foot to the other, was calculated from the forces exerted by each lower limb on the ground measured separately, as proposed by Bastien et al. [45]. *W*_int,dc_ was also divided by TM to obtain mass-relative *W*_int,dc_ (*W*_int,dc.TM_, in J.kg^−1^.m^−1^). Finally, locomotor efficiency (in %) was calculated as the ratio of *W*_ext_ plus *W*_int,dc_ to net *C*_W_ (all in J.m^−1^).

### Statistical Analyses

All descriptive data are presented as mean ± SD. Normal distribution of the data was checked by the Shapiro-Wilk normality test and variance homogeneity between samples was tested by the F-Snedecor test. When conditions of t-test and analysis of variance (ANOVA) application were respectively met, each variable studied was compared (i) between the different times of measurements (i.e. PRE vs. POST) using paired t-tests for the NM data, or (ii) in the three different conditions of equipment across times of measurement (time **×** equipment) using two-factor within subjects ANOVAs for the data of locomotion. Newman-Keuls multiple comparison post-hoc tests were used to determine between-means differences if the ANOVA revealed a significant main effect. For the few NM variables that did not meet normality (i.e. KE MVC, *SOL* M-wave peak-to-peak duration, and PF %VA), Wilcoxon tests were used. Statistical significance was accepted at *P*<0.05. Effect size was calculated using Cohen’s d by dividing the mean difference between PRE and POST (in absolute value) by the between-subject standard deviation at PRE [46]. Effect size was therefore used and considered as a supplementary index of the importance of the effect for the variables showing significant differences or statistical trends between PRE and POST. A Cohen’s d value of 0.2 was considered as a small effect, a value of 0.5 was considered as a moderate effect, and a value of 0.8 was considered as a large effect [46].

## Results

### Heart Rate, Global Exertion and Perceived Fatigue

The mean HR over the first 4-h road march was 139±18 bpm (which corresponded to 73.1±7.3% of HR_max_) and ranged from 90±17 to 171±17 bpm (47.0±7.5% to 90.1±6.6% of HR_max_). Over the 14-h “battle” phase, i.e. the entire SMM duration (21 h) minus the 7 hours (4 h + 3 h) of road marches, it was 91±16 bpm (45.3±4.9% of HR_max_) and ranged from 55±11 to 156±13 bpm (26.4±3.9% to 82.8±5.7% of HR_max_). Finally, over the 3-h return road march, the mean HR was 114±17 bpm (59.7±5.6% of HR_max_) and ranged from 80±19 to 149±15 bpm (41.8±7.7% to 78.3±6.6% of HR_max_). The exertion (RPE) represented by the entire SMM was 16.7±2.4 on the 6–20 Borg scale, which corresponded to a “very hard effort”. Individual RPE values ranged from 13 (n = 1, “somewhat hard effort”) to 20 (n = 2, “exhausting effort”). Last, the instant-sensation of general fatigue (RPF) was significantly increased from PRE to POST (*P*<0.01, d = 3.47). RPF were 8.3±2.2 (corresponding to “very very low fatigue”) and 15.9±2.1 (“very high fatigue”) at PRE and POST, respectively.

### Neuromuscular Fatigue and its Central and Peripheral Components

**Figure 2:**
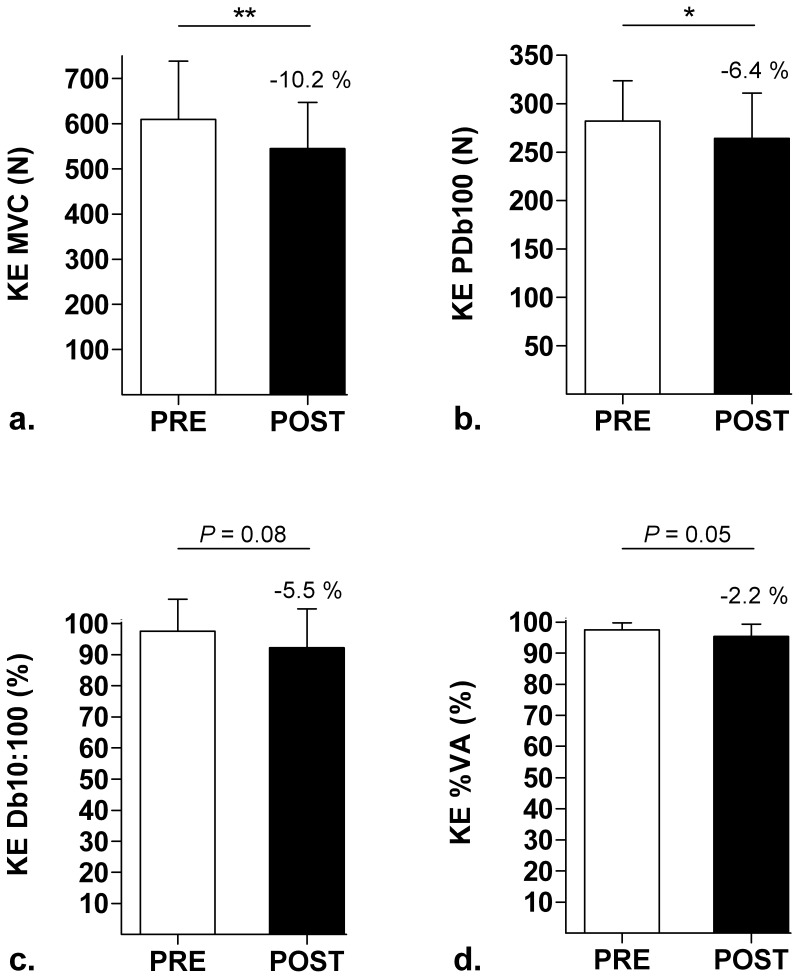
Neuromuscular parameters measured in the knee extensors (KE) before (PRE) and after (POST) the mission. a. Maximal voluntary contraction (MVC); b. Potentiated high-frequency doublet (PDb100); c. Ratio of paired stimulation peak forces at 10 Hz to 100 Hz (Db10∶100); d. Voluntary activation level (%VA). *P<0.05, **P<0.01.

**Figure 3:**
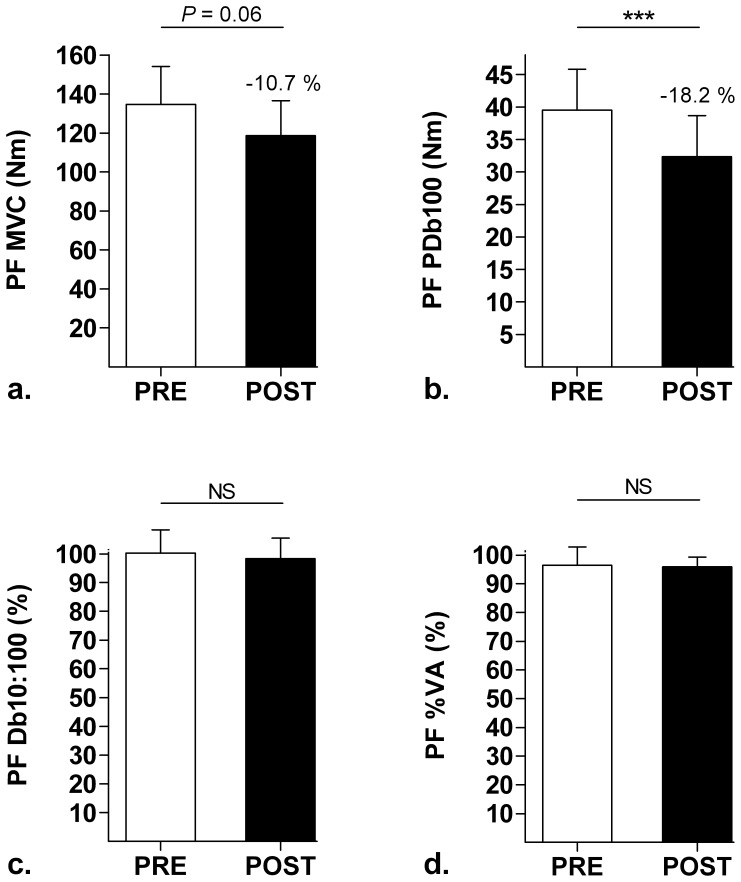
Neuromuscular parameters measured in the plantar flexors (PF) before (PRE) and after (POST) the mission. a. Maximal voluntary contraction (MVC); b. Potentiated high-frequency doublet (PDb100); c. Ratio of paired stimulation peak forces at 10 Hz to 100 Hz (Db10∶100); d. Voluntary activation level (%VA). ***P<0.001.

As shown in figures 2 and 3, MVC declined significantly by −10.2±3.6% for KE (*P*<0.01, d = 0.50) and a strong trend (−10.7±16.1%) was observed for PF (*P* = 0.06, d = 0.82) after the SMM. Concerning the peripheral aspect of fatigue induced by the SMM, PDb100 decreased significantly for KE (−6.39±8.01%, *P*<0.05, d = 0.43) and even more for PF (−18.2±9.8%, *P*<0.001, d = 1.14). A trend towards Db10∶100 decline (i.e. LFF index) was observed for KE (−5.46±9.35%, *P* = 0.08, d = 0.53) but not for PF (*P* = 0.21) after the SMM. There was no significant correlation between KE and PF changes in MVC or PDb100. Table 1 shows the characteristics of the mechanical (KE and PF) and EMG (*VL* and *SOL*) responses to single electrical stimuli of the femoral and tibial motor nerves to the relaxed muscle. In particular, this table shows that the SMM induced considerable effects on the potentiated Pt and CT for both KE and PF. Concerning the central aspect of fatigue, the SMM did not induce change in %VA for PF (*P* = 0.73), but a decreasing trend was observed for KE (−2.18±2.96%, *P* = 0.05, d = 0.96). RMS.M^−1^ did not change significantly from PRE to POST for either KE (from 6.89±2.81% to 6.17±2.01%, *P* = 0.14) or PF (from 3.58±0.63% to 3.58±1.09%, *P* = 0.99).

**Table 1:** Potentiated peak twitch of knee extensors (KE) and plantar flexors (PF), and M-wave characteristics of *vastus lateralis* (*VL*) and *soleus* (*SOL*) muscles, before (PRE) and after (POST) the Simulated Military Mission.

Parameters and muscles	PRE	POST	*t-test P* values	% Change	Cohen’s d
Potentiated peak twitch					
KE (N)	180±32	160±35	**<0.01**	**−10.9**±**9.6**	**0.60**
PF (Nm)	25.6±5.6	20.9±4.6	**<0.001**	**−18.3±9.5**	**0.84**
Contraction time					
KE (ms)	106±4	100±8	*0.07*	−*5.8*±*9.2*	*1.48*
PF (ms)	94.3±16.6	82.8±8.1	**<0.01**	**−10.9±9.2**	**0.69**
Half relaxation time					
KE (ms)	89.7±18.2	84.0±17.4	0.53	−3.6±23.1	0.31
PF (ms)	109±14	100±18	0.17	−7.7±18.8	0.68
M-wave peak-to-peak amplitude					
* VL* (mV)	11.9±3.7	12.1±3.1	0.63	+4.8±15.6	0.06
* SOL* (mV)	7.8±2.9	9.2±2.6	**<0.01**	**+22.2±21.2**	**0.48**
M-wave peak-to-peak duration					
* VL* (ms)	9.8±2.3	9.8±1.4	0.89	+1.4±13.0	0.03
* SOL* (ms)	3.1±1.2	3.1±1.4	0.99	**−**1.2±13.2	0.00

Data are presented as mean ± SD.

### Walking Energetics and Mechanics

Metabolic and mechanical parameters of walking, taking into account the main effects of SMM-related fatigue, acute load carriage and their interaction are presented in tables 2 and 3, respectively. ANOVAs showed that acute military equipment (i.e. load) carriage induced significant increases in both gross and net *C*_W_ (*P*<0.0001). A trend toward increased net *C*_W.TM_ was also observed when load increased (*P* = 0.098) while gross *C*_W.TM_ decreased significantly in this condition (*P* = 0.012). Concerning the mechanics of walking, all spatio-temporal parameters were altered by acute load carriage (all *P*<0.05) and both absolute and mass-relative *W*_int,dc_ and *W*_ext_ increased (all *P*<0.01). However, the inverted pendulum recovery of mechanical energy and the locomotor efficiency did not change while carrying loads. In contrast to these acute effects of load carriage, neither the time between PRE and POST (i.e. the fatigue induced by the SMM), nor the interaction between time and equipment (i.e. fatigue **×** load) had a significant effect on the parameters describing subjects’ walking energetics and mechanics.

**Table 2:** Energy cost of walking in Sport (SP), Battle (BT) and Road March (RM) conditions, before (PRE) and after (POST) the Simulated Military Mission.

	PRE			POST			ANOVA *P* values		
Parameters	SP	BT	RM	SP	BT	RM	T	E	T×E
Gross *C*_W_ (J.m^−1^)	271±36£	336±58	369±55	278±48#	337±64	355±68	0.283	**<0.0001**	0.529
Gross *C*_W.TM_ (J.kg^−1^.m^−1^)	3.30±0.47	3.22±0.50	3.07±0.38	3.43±0.54$	3.28±0.60	3.00±0.50	0.121	**0.012**	0.484
Net *C*_W_ (J.m^−1^)	144±37£	205±56	239±70	150±42#	210±56	228±60	0.578	**<0.0001**	0.529
Net *C*_W.TM_ (J.kg^−1^.m^−1^)	1.74±0.44	1.97±0.46	1.98±0.44	1.85±0.45	2.04±0.52	1.92±0.45	0.303	*0.098*	0.596

Values are presented as mean ± SD. ANOVA *P* values represent the main effects of time (T) and equipment (E), and the interaction effect (T×E). Post-hoc results are presented as:

£ SP< BT < RM;

# SP< BT = RM;

$ SP> RM.

*C*_W_: energy cost of walking; *C*_W.TM_: energy cost of walking normalized to the total mass (TM) moving on the treadmill, i.e. subject plus equipment.

**Table 3:** Spatio-temporal and kinetic parameters of walking in Sport (SP), Battle (BT) and Road March (RM) conditions, before (PRE) and after (POST) the Simulated Military Mission.

	PRE			POST			ANOVA *P* values		
Parameters	SP	BT	RM	SP	BT	RM	T	E	T×E
Step Frequency (Hz)	1.73±0.09$	1.73±0.07	1.69±0.07	1.75±0.09	1.76±0.10	1.71±0.07	0.147	**0.012**	0.864
Duty factor (%)	64.9±0.7£	66.3±0.8	66.9±0.9	65.1±0.4£	66.4±0.8	67.4±1.2	0.211	**<0.0001**	0.299
Double support duration (%)	29.6±1.4£	32.6±1.6	33.8±1.7	30.3±0.9£	32.6±1.7	35.0±2.3	0.196	**<0.0001**	0.140
*W*_ext_ (J.m^−1^)	19.4±3.6£	28.5±4.8	31.9±7.0	19.2±3.3£	26.6±4.1	31.4±5.3	0.117	**<0.0001**	0.187
*W*_ext.TM_ (J.kg^−1^.m^−1^)	0.228±0.041#	0.267±0.041	0.261±0.060	0.231±0.046#	0.254±0.038	0.262±0.050	0.557	**0.004**	0.238
Recovery (%)	72.2±3.9	70.8±3.1	71.5±3.9	71.9±4.6	72.3±2.8	71.7±3.0	0.496	0.874	0.174
*W*_int,dc_ (J.m^−1^)	11.5±2.8£	15.9±4.7	19.7±5.0	11.2±3.1£	15.2±3.9	19.9±4.4	0.695	**<0.0001**	0.587
*W*_int,dc.TM_ (J.kg^−1^.m^−1^)	0.134±0.030£	0.147±0.041	0.161±0.041	0.133±0.032&	0.144±0.035	0.166±0.038	0.957	**<0.0001**	0.559
Locomotor efficiency (%)	22.6±7.9	23.9±10.8	22.9±8.9	19.8±4.4	20.7±6.6	23.9±7.9	0.225	0.362	0.300

Values are presented as mean ± SD. ANOVA *P* values represent the main effects of time (T) and equipment (E), and the interaction effect (T×E). Post-hoc results are presented as:

$ SP = BT > RM;

£ SP< BT < RM;

# SP< BT = RM;

& SP = BT < RM.

*W*_ext_: external mechanical work; *W*_ext.TM_: external mechanical work normalized to the total moving mass (TM), i.e. subject + equipment; Recovery: fraction of mechanical energy of the center of mass recovered via the inverted pendulum mechanism; *W*_int,dc_: mechanical work done by one leg against the other leg during double contact; *W*_int,dc.TM_: mechanical work done by one leg against the other leg during double contact normalized to TM; Locomotor efficiency: ratio of mechanical works (*W*_ext_ and *W*_int,dc_) to net *C*_W_.

## Discussion

The Main Purpose of the Present Study was to Investigate the combined effects of heavy load carriage and exercise of extreme duration on NM fatigue. Specifically, we hypothesized that both central and peripheral NM function changes due to a 21-h military mission would be higher than after walking with load carriage for intermediate durations, i.e. up to ∼3 h [18], [19], but lower (especially at the peripheral level) than after ultra-marathon runs lasting up to ∼40 h and inducing extensive muscular damage [21], [22]. The main results of this study are: (i) MVC declined by ∼10% after the SMM and the origin of fatigue was essentially peripheral for both KE and PF, both of which are major power generators in human locomotion, (ii) a trend toward moderate LFF was detected for KE muscles, suggesting the presence of muscular mechanical and metabolic disturbances, and (iii) NM function changes were concomitant with severe subjective fatigue but the latter was not coupled with the central NM mechanisms. The secondary aim of this study was to investigate the effects of extreme-duration heavy load carriage on the energy cost, mechanical work and spatio-temporal pattern of walking. In particular, we hypothesized that the fatigue induced by a 21-h SMM would affect the biomechanics of walking. The results indicated that neither walking energetics, nor walking mechanics were altered by SMM-related fatigue. Taken together, these results bring the first insight into the physiological and biomechanical consequences of exercises of extreme duration with heavy load carriage. Overall, it appears that extreme-duration heavy load carriage induced modest consequences in experienced walkers/carriers, contrary to what was expected from both NM and locomotor standpoints.

### Exertion and Perceived Fatigue Due to the Military Mission

HR data monitored over the entire SMM showed that the most severe period of the mission was the first 4-h march (∼73±7% of HR_max_), very likely due to the long and sometimes steep uphill sections (figure 1) with heavy load carriage (∼43 kg). In contrast, the 14-h-battle phase was the least demanding period of the SMM (∼45±5% of HR_max_), notably because of the two 3-h sleeping periods permitted during this phase. Nevertheless, HR peaked around 83±6% of HR_max_ during the battle phase, showing that the recons and patrols were quite intense. Finally, although the 3-h return march was performed at a high speed (∼5.5 km.h^−1^ on average) considering the load carried and the preceding efforts, the major presence of downhill sections resulted in a relatively moderate HR (∼60±6% of HR_max_). The overall profile of the SMM (figure 1) was consistent with the observations reported in the military literature, namely the major presence of endurance activities combined with short periods of intense efforts [10].

After exercise, the subjects rated the entire SMM as “very hard” (16.7±2.4 on the 6–20 Borg’s scale) despite their substantial experience with this type of effort. Moreover, their RPF increased significantly from PRE (8.3±2.2, “very very low fatigue”) to POST (15.9±2.1, “very high fatigue”; *P*<0.01, d = 3.47). Interestingly, the subjects’ sensation of fatigue increased by almost 100% from PRE to POST, whereas their MVC (i.e. the global index of NM fatigue) declined only moderately (∼10%, see below). However, it is likely that the peripheral NM fatigue observed in this study and the environmental stressors induced by the mission (e.g. sleep and food rhythms disturbances), which are known as drivers of subjective fatigue [32], [47], [48], have contributed to this result.

### Neuromuscular Fatigue Induced by the Military Mission

#### Comparison with ultra-endurance exercise without load carriage

As hypothesized, the SMM, which mainly consisted of walking for extreme duration with heavy loads, induced lower NM function alterations than ultra-marathons, which consist of running and high-speed walking for extreme duration with very light equipment. Indeed, the average extent of force loss from PRE to POST (∼10% for both KE and PF, see figures 2 and 3) was 3 to 4 times lower in the present study than previously observed after 24-h [21] and mountain [22] ultra-marathons for the same muscle groups. Moreover, central mechanisms, which are known as major components of NM fatigue after ultra-endurance runs [21], [47], [49], were only slightly affected in KE after the SMM, as shown by the −2.2±3.0% decline in %VA (*P* = 0.05, d = 0.96). A notable difference between ultra-marathons and the SMM is that in the former subjects perform ultra-endurance exercise as fast as they can, while the military subjects of the present study followed instructions and velocity plans. Therefore, results about MVCs and %VA after the SMM should be considered, among others, in light of this important difference with ultra-marathons. Nevertheless, the present results were in line with previous studies showing that KE are more prone to central fatigue than PF [21], [22].

The results of this study indicate that the impact of extreme-duration heavy load carriage on peripheral fatigue should not be underestimated. Indeed, contrary to what was hypothesized, peripheral NM function alterations were large after the SMM, i.e. almost as large as in ultra-marathons, especially for PF muscles. For instance, PDb100 decreased by −6.4±8.0% for KE (*P*<0.05, d = 0.43) and −18.2±9.8% for PF (*P*<0.001, d = 1.14) after the SMM, while the mountain ultra-marathon study reported mean declines of about −12% for KE and −20% for PF [22]. Furthermore, single stimuli to the relaxed muscles showed large decreases in potentiated Pt and CT after the SMM (table 1), corresponding to specific alterations comparable to those reported after ultra-endurance runs, especially for PF [22], [49]. This general result regarding peripheral fatigue was not expected since, for conditions of duration and environment comparable to the SMM, extreme ultra-marathons are associated with much larger impacts underwent by the locomotor system at each step [34]. However, the fact remains that load conditions are very different between these two types of exercise. The severe loads carried during the SMM may therefore have contributed to the substantial peripheral fatigue after the SMM. Finally, the presence of LFF in KE muscles after the SMM (Db10∶100 declined by −5.5±9.3%, *P* = 0.08, d = 0.53) suggests specific failures in excitation-contraction coupling (i.e. reduction in Ca2+ release and/or decreased myofibrillar Ca2+ sensitivity [13]) and muscular damage [20]. Similarly, for PF muscles the decrease in paired stimuli peak force evoked at 10 Hz, yet in the same proportion as PDb100 since Db10∶100 did not change, suggests alterations inside the muscle cell [15]. This trend toward LFF after the SMM therefore indicates that the belief that low-speed locomotion is insufficient to promote the development of LFF [21] is not valid when subjects carry heavy loads, especially in KE muscles, which were more prone to LFF in the present study.

Unlike ultra-marathons after which lower amplitude and longer duration of M-wave were found [22], the SMM induced a 22.2±21.2% increase in the *SOL* M-wave amplitude (*P*<0.01, d = 0.48; see table 1). This result constitutes the most striking difference in peripheral NM function alterations between extreme-duration heavy load carriage and extreme-duration runs. This also suggests that muscle excitability was preserved for both *VL* and *SOL* after the SMM. Therefore, processes located distal to the action potential propagation/transmission, involving Ca^2+^ and/or cross-bridge kinetics [15], were implicated in the reduction of the evoked mechanical responses, in line with the trend toward LFF discussed previously.

#### Comparison with load-carrying exercise of moderate duration

We are not aware of any data regarding PF NM fatigue after load-carrying exercise. It is unfortunate since PF are more solicited than KE during walking, at least on flat terrain. The results of this study (i.e. PF MVC decline of ∼11% associated with large peripheral fatigue after the 21-h SMM) therefore constitute to our knowledge the first data for future discussions about this muscle group. Concerning KE, it was surprising to see that the 21-h SMM induced an overall fatigue (MVC decrease of ∼10%, figure 2) similar to that reported after much shorter load-carrying exercises. Indeed, after 12.1-km road marches performed at 4 km.h−1 with loads up to 27 kg, Clarke et al. [18] observed peak torque declines of about 8% in military subjects. Blacker et al. [19] even showed MVC decrease of ∼15% (i.e. larger than in the present study) after bouts of 2-h treadmill walking performed at 6.5 km.h−1 with a 25-kg backpack. In addition, beyond this observation about force loss, they also pointed out KE central activation deficit (4.2%) and LFF (4.5%) [19], which represent central and peripheral NM function changes of KE similar to the present ones. This comparison therefore contradicts our initial hypothesis that the SMM would induce larger NM function alterations than load-carrying exercises of intermediate durations.

Although, at first glance, this finding could seem surprising because of the differences among studies in terms of exercise durations, environmental stressors and loads carried [18], [19], factors such as subjects’ experience and mission profile should be considered. In fact, Blacker et al. [19] studied recreational hikers while the present study investigated very experienced soldiers highly trained in load carriage exercise [50]. Furthermore, when looking at the SMM profile (figure 1), the distribution of the sleep, rest and rationing periods throughout the military mission likely allowed infantrymen to recover from or limit NM fatigue. Investigating the NM function of experienced soldiers after a similar mission without rest and sleep would therefore be interesting for future research. Nevertheless, the present results indicate that the typical management of soldiers’ efforts during missions seems beneficial to preserve their NM function.

#### Effects of load and military mission-related fatigue on soldier locomotion

The results regarding the effects of acute military load carriage on locomotion (tables 2 and 3) were in line with our recent study [4]. Briefly, the energy cost, mechanical work and spatio-temporal pattern of walking were significantly altered by load carriage [3], [4], [5], [6]. Only the results relating to the effects of the SMM are discussed below.

Contrary to our initial hypothesis, neither the fatigue induced by the SMM, nor the interaction between SMM-related fatigue and acute load carriage induced significant changes in walking energetics or mechanics. Of these, the absence of fatigue effect (vs. interaction effect) is the most surprising result, especially from a mechanical standpoint [30], [31]. Indeed, recent studies have shown that foot pressures, gait kinematics, peak ground reaction forces and loading rates are affected by fatiguing laboratory protocols and prolonged outdoor walking [25], [27], [28], [29], [30], [31]. Consequently, after such an extreme-duration exercise with severe loads, we expected changes in the walking gait. Nevertheless, the choice of mechanical parameters may partly explain the lack of alteration in the present study. Indeed, among the researchers that have investigated the effects of fatigue on the spatio-temporal pattern of walking, Qu & Yeo [30], for instance, detected alterations but the significant differences they observed only concerned gait variability. Moreover, the other studies that have explored the effects of fatigue on walking mechanics mainly focused on plantar pressures [25], [28] and peak forces during foot-ground contact [31], which represent different mechanical aspects of human walking than the absolute spatio-temporal parameters and mechanical work studied here (see e.g. ref. [29]).

Nevertheless, in light of the relatively moderate NM function alterations observed after the SMM, it is also likely that our experienced soldiers were able to compensate for this KE and PF fatigue by biomechanical and NM adjustments [50]. Moreover, even if their sensation of general fatigue was high, the subjects did not report or mention lower-limb pain, which could have resulted in spatio-temporal or kinetic locomotor adaptations after the SMM, as shown after extreme runs [34], [51]. Such compensation for NM fatigue would however be unlikely in subjects performing an isokinetic fatiguing protocol inducing KE MVC decreases of 60% for instance [27]. Consequently, the present results raise the question of the scope of fatiguing laboratory protocols, which may induce artificial effects as compared with field protocols that reflect the reality of exercise [32]. Indeed, if we consider our subjects’ locomotor responses after the SMM as representative of those of soldiers/walkers in real mission/trekking contexts, we can conclude that walking pattern and mechanical work do not appreciably change after overall heavy load carriage exercises of extreme-duration. This could also suggest that factors other than biomechanical and NM ones may better explain the accident-related musculoskeletal injuries reported after missions/trekking [7]. Decreased attention and cognitive fatigue are potential explanations but further study is necessary.

One possible limitation of the present study was the absence of a control group to rule out the potential effects of sleep rhythm disturbance on the NM function (i.e. only two 3-h sleeping periods separated by a 2-h period were allowed). However, in a previous study from our research group [21], we showed that after 24 h of total sleep deprivation the subjects did not show any NM change. Thus, we assumed that this methodological choice did not alter the present data. Incidentally, the study of Rodgers et al. [48] showed no NM alteration in control groups subjected to 48 h without sleep, which reinforces our assumption on this point. Another potential limitation was the delay between the end of the SMM and the walking measurements, due to the NM function evaluation, which may have permitted some recovery. Nevertheless, when subjects present moderate NM function alterations such as those observed in the present study, metabolic factors are minimally involved in fatigue. Therefore, this delay in performing the measures does not seem critical (especially as the NM evaluation involved muscular contractions) and we can reasonably assume that this methodological choice did not change the results obtained in walking. Finally, although the SMM was designed to represent a military mission, it is accepted that such a simulation differs from the operational reality (i.e. absence of stress, fear, or operational goals). Thus, the mission environment represented here must be considered as a military-like physical context rather than an operational theater-like one. However, we are convinced that such a real-world approach remains more representative and beneficial than fatiguing laboratory protocols.

### Conclusion

The results of the present study showed that the central and peripheral NM function alterations were not larger after the 21-h military mission than after load-carrying exercises lasting from 2 to 3 h, contrary to our first hypothesis. Moreover, the NM function changes were lower after the 21-h military mission performed with heavy load carriage than after ultra-marathon runs, in accordance with our second hypothesis. Consequently, extreme-duration heavy load carriage induced overall moderate central and peripheral fatigue in experienced carriers for the two major muscle groups implicated in human walking (i.e. KE and PF). The lack of substantial central fatigue observed in the present study might be attributed, at least in part, to the beneficial resting and sleep times realistically distributed throughout exercise. Thus, NM fatigue was mainly attributable to peripheral alterations although it came with a significant perceived fatigue. It is therefore recommended to exploit each minute of resting/sleep periods to recover from and/or limit fatigue during such exercises, as observed in the very experienced soldiers of this study.

***§ 50 -*** Concerning the secondary aim of the present study, contrary to our third hypothesis, results indicated that the mechanical and metabolic parameters of walking do not appreciably change after an exercise lasting 21 h and involving severe load carriage. Therefore, assuming a link between perceived fatigue and attention/cognitive capabilities, it is expected that a large sensation of fatigue may decrease carriers’ attention and, thus, partly explain the accident-related injuries arising at the end of prolonged load-carrying exercise (since the objective parameters reported here do not allow explanation of these kind of injuries).
